# Miscellaneous

**Published:** 1885-11

**Authors:** 


### MISCELLANEOUS.

"WHAT makes the sea salt?" said the teacher. And young America shouted, "The codfish that are in it."

A NEW YORK paper speaks of a man who was "beaten in three suits," which reminds one of the old-time schoolboy who used to pad his trousers in anticipation of a thrashing.

"IT is truly amusing," says the London *Truth*, "to see the assembled wiseacres of the British Institution making discoveries in canine intelligence which must have been common knowledge to dog-fanciers in the days of Nimrod. Sir John Lubbock's learned poodle is a fool beside a little performing mongrel which I have lately seen at one of the south-coast watering places. Any spectator was invited to show this little animal his watch, when the dog, after studying the face for a moment, would proceed to tell the time by selecting the proper figures from a row of Arabic (not Roman) numerals plgced before him. Again, professor Flower's eminently Scotch tyke who refused to go for a walk on the 'Sawbath' is very little ahead of the dog whom most of us must have seen or heard of, who loses all desire to go out on Sunday morning upon being shown a prayer-book In the same way, I have heard of a university dog—probably not a unique specimen—who, though always frantic to start out the moment his master took up his hat, never offered to move when the head covering was a college cap.

RESPECTING THE SABBATH.—The people of a New Hampshire town are so fearfully lazy that, when the wife of a minister who had just settled in that town asked a prominent citizen if the inhabitants generally respected the Sabbath and refrained from business, he replied: "Confound it, ma'am, they don't do enough work in a whole week to break the Sabbath if it was all done on that day."

BROKE THE CAR-STRING.—As a train was approaching Cleveland it parted in the middle, and the bell-rope snapped off like a thread, the end of it striking an old lady on her bonnet.

"What is the matter?" she exclaimed.

"Oh, the train's broke in two," replied a gentleman who sat in the next seat.

"I should say so," the old lady said, looking at the broken bell-cord. "Did they s'pose a trifling little string like that would hold the train together?"

